# Agrobacterium-derived DNA sequences in phylogenetic studies of plants

**DOI:** 10.18699/vjgb-25-93

**Published:** 2025-10

**Authors:** T.V. Matveeva, P.M. Zhurbenko, G.V. Khafizova, A.D. Shaposhnikov, R.R. Zhidkin, A.V. Rodionov

**Affiliations:** Saint-Petersburg State University, St. Petersburg, Russia; Komarov Botanical Institute of the Russian Academy of Sciences, St. Petersburg, Russia; University of Houston, Houston, United States; Saint-Petersburg State University, St. Petersburg, Russia; Saint-Petersburg State University, St. Petersburg, Russia; Saint-Petersburg State University, St. Petersburg, Russia Komarov Botanical Institute of the Russian Academy of Sciences, St. Petersburg, Russia

**Keywords:** agrobacterium-mediated transformation, cellular T-DNA, phylogenetics, Nicotiana, Camellia, Vaccinium, Arachis, агробактериальная трансформация, клеточная Т-ДНК, филогенетика, Nicotiana, Camellia, Vaccinium, Arachis

## Abstract

One of the main methods for obtaining transgenic plants is Agrobacterium-mediated transformation. This process relies on the ability of certain soil bacteria, specifically from the genera Agrobacterium and Rhizobium, to transfer and integrate a fragment of their plasmid into the chromosome of the recipient plant. This transferred DNA is referred to as T-DNA. Laboratory studies have demonstrated that whole plants can be regenerated from transgenic cells. It soon became evident that similar processes occur in nature, leading to the emergence of naturally transgenic plants, or natural GMOs. Thus, naturally transgenic plants possess homologues of the T-DNA genes from agrobacteria in their genomes (cellular T-DNA, or cT-DNA). These sequences are inherited through multiple sexual generations and retain their functionality. Furthermore, the potential for using newly acquired plant sequences in phylogenetic studies has been established, as cT-DNAs are clearly defined, highly specific, and recognizable DNA fragments that differ from typical plant DNA sequences. They are not found in untransformed ancestors, and their integration at specific chromosomal sites marks a monophyletic group of species. This review highlights the diversity of cellular T-DNAs and their potential use as phylogenetic markers. It includes a description of the main methodological approaches to such studies and discusses specific examples that clarify controversial points in the phylogeny of the genera Nicotiana, Camellia, Vaccinium, and Arachis. An important aspect of phylogenetic analysis based on cT-DNA is the assembly of individual alleles, which enables the tracking of interspecific hybridization events. This approach demonstrated the incomplete process of speciation within the Thea section of the genus Camellia and confirmed the role of interspecific hybridization in the breeding of North American blueberries. The review also addresses the dating of transformation events based on cT-DNA, which are organized in the form of imperfect repeats, as well as the application of phylogenetic studies to investigate the biodiversity of agrobacterial T-DNA genes.

## Abstract (ru)

Агробактериальная трансформация – наиболее распространенный метод получения трансгенных растений. Метод основан на способности определенных почвенных бактерий родов Agrobacterium и Rhizobium переносить и интегрировать в хромосому растения-реципиента фрагмент своей плазмиды. Этот фрагмент получил название Т-ДНК (transferred DNA – переносимая ДНК). В лабораторных условиях было показано, что из трансгенных клеток можно регенерировать целые растения. Вскоре стало ясно, что подобные процессы происходят и в природе, приводя к появлению природно-трансгенных растений или природных генно-инженерно-модифицированных организмов (ГМО). Таким образом, природно-трансгенными называются растения, у которых в геномах присутствуют гомологи генов Т-ДНК агробактерий (клеточные Т-ДНК, клТ-ДНК). Эти последовательности наследуются в ряду половых поколений и сохраняют свою функциональность. Кроме того продемонстрирована возможность использования новоприобретенных растениями последовательностей в филогенетических исследованиях, поскольку клT-ДНК являются четко определенными, высокоспецифичными и узнаваемыми фрагментами ДНК, не похожими на последовательности ДНК растений. Они не встречаются у нетрансформированных предков, и их интеграция в определенном хромосомном сайте маркирует монофилетическую группу видов. В представленном обзоре освещены вопросы разнообразия клТ-ДНК, возможности их применения как филогенетических маркеров, в том числе описаны основные методические подходы таких работ, на конкретных примерах рассмотрены возможности уточнения спорных моментов в филогении родов Nicotiana, Camellia, Vaccinium и Arachis. Важным моментом филогенетического анализа на основе клТ-ДНК является реконструкция отдельных аллелей. Это дает возможность отслеживать факты межвидовой гибридизации. Именно этот подход позволил продемонстрировать незавершенный процесс видообразования в пределах секции Thea рода Camellia, а также подтвердил использование межвидовой гибридизации в ходе селекции североамериканских голубик. В обзоре рассмотрены вопросы датировки событий трансформации на основе клТ-ДНК, организованных в виде несовершенных повторов, а также использования филогенетических исследований при изучении вопросов биоразнообразия генов Т-ДНК агробактериального происхождения

## Introduction

Agrobacterium-mediated transformation is currently the most common method for producing transgenic plants to meet the needs of agriculture, medicine, veterinary science, and other sectors of the national economy. This method relies on the ability of soil bacteria, specifically Agrobacterium tumefaciens (Smith and Townsend 1907) Conn 1942 (Approved Lists 1980) and Rhizobium rhizogenes (Riker et al., 1930) Young et al., 2001, to transfer a fragment of their plasmid and integrate it into plant DNA (Schell, Van Montagu, 1977; Bahramnejad et al., 2019). The transferred sequences are referred to as T-DNA (transferred DNA).

Under natural conditions, the transfer and integration of T- DNA into the host plant chromosome typically stimulate the growth of transgenic tissue due to the expression of T-DNA genes that regulate morphogenesis (Nester, 2015). However, the regeneration of fully transgenic plants from such tissues has also been documented (Tepfer, 1990; Christey, 2001). Numerous observations suggest that this process can occur in nature without human intervention, resulting in the emergence of plants containing bacterial sequences in their genomes, which can be passed down through successive sexual generations (Matveeva, 2021). The first naturally transgenic plants were identified among species of the genus Nicotiana in 1983 (White et al., 1982, 1983), and currently, dozens of genera of dicots have been documented to contain naturally transgenic species (Matveeva, Otten, 2019; Matveeva, 2021). T-DNA found in the genomes of natural GMOs (nGMOs) is referred to as cellular T-DNA (cT-DNA) (Matveeva, 2021). Comparing the number of species with sequenced genomes to the number of nGMOs among them, we find that traces of Agrobacterium-mediated transformation have been preserved in seven percent of dicotyledonous plant species (Matveeva, Otten, 2019). This provides scientists with extensive material to study the functions of bacterial genes in plants and their evolutionary trajectories

Additionally, interesting data have been obtained using T- DNA as a phylogenetic marker, as newly acquired sequences offer several advantages for studying the origin and evolution of nGMO species (Matveeva et al., 2011; Chen et al., 2022; Zhidkin et al., 2023; Bogomaz et al., 2024). cT-DNA are clearly defined, highly specific, and recognizable DNA fragments that differ from plant DNA sequences. They are absent in untransformed ancestors, and their integration at specific chromosomal sites marks a monophyletic group of species. Typically, cT-DNAs consist of single insertions, which is a significant advantage over classical nuclear markers. Furthermore, cT-DNAs can be quite long and ancient, accumulating single nucleotide substitutions (SNS), which allows for the construction of phylogenetic trees. The probability that the same T-DNA sequence will integrate into the genomes of two independent phylogenetic branches at the same target site with identical boundaries seems unlikely, but not impossible. This makes T-DNA insertions, along with transposon insertions into the genome, extremely important synapomorphies (Shedlock, Okada, 2000; Doronina et al., 2022) and, therefore, powerful tools for systematics. Their genome-wide analysis may also help identify the causes of phylogenetic signal conflicts should they arise (Kuritzin et al., 2016). Lastly, repetitive sequences in cT-DNA can be used to estimate the time since transformation or serve as relative time markers (Chen et al., 2022).

## T-DNA structures

The physical structure of T-DNA varies among plasmids from different Agrobacterium strains and can be classified based on the number of plant-transferable fragments encoded in a single plasmid. T-DNA can be continuous, as seen in mannopine, mikimopine, and cucumopine strains (Jouanin, 1984; Hansen et al., 1991). This continuous fragment of DNA is flanked by border sequences at both ends, as exemplified by plasmid pRi8196. In contrast, other plasmids, such as pRiA4, have T-DNA divided into two segments: TL-DNA and TR-DNA (White et al., 1985), separated by a non-plant-transferable DNA region of about 15 kb that acts as a spacer. Regardless of their structural differences, all aforementioned T-DNAs contain genes for the synthesis of opines, which serve as a food source for Agrobacterium, as well as oncogenes, the products of which induce plant cell division

The structures of nGMO cT-DNAs are more diverse, with those containing only opine synthesis genes being the most prevalent. This phenomenon can be attributed to at least three factors. First, in the known T-DNAs, the opine synthesis genes are typically located closer to the right border. During transformation, when single-stranded T-DNA is excised from the plasmid, the VirD2 protein covalently binds to the 5ʹ end of the T-DNA at its right border and subsequently directs the T-strand through the type IV protein secretion system into the plant cell (Gelvin, 2021). Deletions that occur during the integration of T-DNA into the plant chromosome tend to be more extensive at the 3ʹ end than at the 5ʹ end, which is spatially protected by the VirD2 protein (Gelvin, 2021). Secondly, it is plausible that there exist Agrobacterium strains, the T-DNA of which contains only opine synthase genes. Thirdly, it is possible that significant portions of the T-DNA may have been lost after integration into the chromosome during the evolution of the descendants of a natural transformant, retaining only the opine synthesis genes. These genes do not influence morphogenesis as significantly as oncogenes, meaning they do not lead to growth and developmental abnormalities (Matveeva, Otten, 2021).

The other most common structures are extended T-DNA fragments containing both oncogenes and opine synthesis genes. These cT-DNAs are typically represented by inverted imperfect repeats, a feature that can be used to date transformation events. The least common are cT-DNAs containing only oncogenes (Matveeva, Otten, 2019; Matveeva, 2021).

Let us now explore in more detail the issue of dating the emergence of nGMOs throughout evolution

## Approaches to determining the timing of transformation events in nGMOs

The dating of transformation events in nGMOs can be performed using the molecular clock method. The peculiarities of T-DNA integration into the genome of an infected plant lead to the formation of repeating T-DNA sequences or regions (Tzfira et al., 2004; Singer, 2018). These repeats are long cT-DNAs, represented by imperfect inverted repeats or their fragments formed during region deletions or insertions (Matveeva, Otten, 2019; Matveeva, 2020). By analyzing the differences in the nucleotide sequences of these repeats, it is possible to estimate the approximate time of divergence and, consequently, the time of T-DNA integration into the genome of the future nGMO, using the nucleotide substitution coefficient (Gaut et al., 1996).

The transformation time can be calculated using the following formula

**formula. 1.**
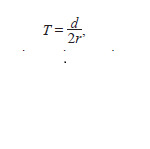
formula1.

where T – the approximate integration time of cT-DNA; d – the ratio of nucleotide differences between two repeats; r – the average nucleotide substitution rate of 6.5 × 10–9 substitutions per site per year (Gaut et al., 1996; Lynch, Conery, 2000). This approach was used to date T-DNA integration in representatives of the Camellia genus, which possess various structural types of cT-DNA (Chen et al., 2022, 2023). The dating revealed that the oldest integration event occurred approximately 7.5 million years ago, while the youngest took place around 0.04 million years ago (Chen et al., 2023). It was clarified through correspondence with the authors that the insertion times reported in the article were calculated using an erroneous formula, resulting in values that were overestimated by a factor of two. This review presents the corrected values.

However, this method has several limitations that hinder its application for determining the integration times of many cT-DNAs. The first limitation is the inability to detect inverted repeats in all cT-DNAs, as most nGMO species do not contain extended cT-DNAs where repeating direct and inverted sequences are typically found (Matveeva, 2021). Secondly, not all extended cT-DNAs contain repeats; for instance, only 6 out of 12 types of cT-DNAs in members of the genus Camellia possess repeats that facilitate the determination of integration time (Chen et al., 2023). An additional critical consideration when selecting sequences for dating is the necessity to exclude the possibility that these repeats formed as a result of other genomic rearrangements, such as the activity of mobile genomic elements

The method of dating cT-DNA should be employed in conjunction with other approaches to accurately assess the nucleotide substitution rate in a particular species and to compare divergence times with closely related taxa for validation. Although this method of dating nGMO transformation requires further development and verification, it offers valuable insights into the evolutionary processes that nGMOs have undergone following Agrobacterium-mediated transformation.

## Multiple transformation events in the evolution of the genus Nicotiana

The study of genetic transformation in plants occurring naturally, without human intervention, began with the species Nicotiana glauca Graham, which was found to contain sequences homologous to agrobacterial T-DNA in its nuclear genome (White et al., 1983). The first identified cT-DNA was designated gT. This sequence was organized as an imperfect inverted repeat and consisted of one copy of the rolB homolog, along with two copies each of the rolC, ORF13, ORF14, and mis homologs (Suzuki et al., 2002). The gT sequence served as a reference point for the search for cT-DNA in the genomes of other Nicotiana L. species (Furner et al., 1986; Intrieri, Buiatti, 2001). To date, 16 naturally transgenic species of this genus have been identified (Otten, 2020). The genus Nicotiana comprises twelve sections (Knapp et al., 2004), six of which have naturally occurring transgenic representatives described.

The search for and analysis of cT-DNA in plant genomes were initially conducted at the level of individual genes (Furner et al., 1986; Meyer et al., 1995; Intrieri, Buiatti, 2001). During this time, efforts were made to reconstruct the phylogenetic relationships between cT-DNA of various tobacco species and T-DNA of Agrobacterium. However, subsequent studies have called some earlier evolutionary models into question. The transition to whole-genome data has enabled researchers not only to search for cT-DNAs and analyze their composition but also to estimate their quantity and localization within the plant genome. The first such analysis was conducted for N. tomentosiformis Goodsp. (Chen et al., 2014), where four distinct cT-DNA types (TA, TB, TC, and TD) were identified (see the Table), differing from the previously studied gT in N. glauca (Suzuki et al., 2002). This finding highlighted the importance of assessing the number of cT-DNAs in the genome and their localization sites as a means to identify the descendants of specific transformation events.

**Table 1.**
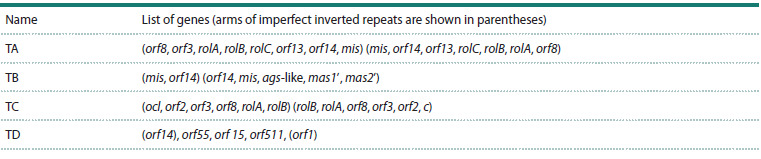
cT-DNA structures in N. tomentosiformis

**Fig. 1.**
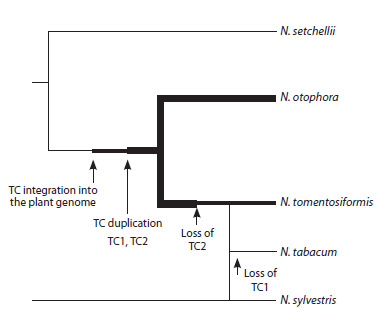
Model of TC cT-DNA evolution in Nicotiana genomes The width of the lines reflects the presence of one or two TC copies in the species genome, or their absence (according to Chen et al., 2018).

Multiple extended cT-DNAs in tobacco species have been used to date transformation events in evolution. In N. tomentosiformis, all four cT-DNAs were shown to result from independent Agrobacterium-mediated transformation events that occurred at different times. The cT-DNA TC was identified as the oldest of the four, with an estimated age of 1 million years (Chen et al., 2014). Later, in the genome of N. otophora Griseb., a species phylogenetically close to N. tomentosiformis, two TC copies were found that differed by 4 % and were located at the same site as the TC of N. tomentosiformis. The common localization site suggests that the cT-DNA TC was most likely acquired by a common ancestral species of N. tomentosiformis and N. otophora (Fig. 1).

During the evolution of this species, a TC duplication likely occurred, leading to the formation of N. otophora, which carries two TC copies in its genome, and N. tomentosiformis, which lost one TC copy during speciation (Chen et al., 2018). Attempts to reconstruct the evolutionary events, including speciation, in the genus Nicotiana are complicated by its characteristic reticulate or mesh-like evolution, marked by partial fusion of ancestral branches and the formation of hybrid forms (Knapp et al., 2004). For example, a wellknown representative of the genus Nicotiana, N. tabacum L. (cultivated tobacco), is an interspecific hybrid of N. tomentosiformis and N. sylvestris Speg. (Yukawa et al., 2006). The genome of N. tabacum contains three of the four cT-DNAs found in N. tomentosiformis (TA, TB, TD), suggesting that Agrobacterium-mediated transformation events in N. tomentosiformis preceded the speciation of N. tabacum (Chen et al., 2014). This assumption is supported by flow cytometry and genomic hybridization estimates, indicating that the age of N. tabacum is less than 600 years (Leitch et al., 2008). The TC cT-DNA, a copy of which is present in the genome of the ancestral species N. tomentosiformis, was likely lost during the speciation of N. tabacum (Chen et al., 2014) (Fig. 1). Using TC cT-DNA dating data and speciation time estimates, it is possible to trace the TC path from its initial transfer into the plant genome to its loss or consolidation in various species that originated from the ancestral form at different stages of the genus’s evolution.

All the representatives described above belong to the Tomentosae and Nicotiana sections. For representatives of the Noctiflorae section (N. glauca, N. noctiflora Hook.), three different cT-DNAs acquired through independent transformation events have also been documented (one insertion in the genome of N. glauca and two in N. noctiflora) (Khafizova et al., 2023). However, additional naturally transgenic representatives with sequenced genomes are needed to elucidate the phylogenetic relationships in this branch.

So, the degree of study of the taxon is directly related to the availability of material and its economic significance. The intraspecific diversity of cultivated tobacco is also of interest; therefore, in the next section, we will focus on the application of cT-DNA in the study of this topic.

## Intraspecific variability of cT-DNA in tobacco

Nicotiana tabacum, or cultivated tobacco, is an allotetraploid formed through the interspecific hybridization of N. tomentosiformis and N. sylvestris (Yukawa et al., 2006). There are tens of thousands of existing varieties and species of N. tabacum, though the relationships among them have not been fully established (Moon et al., 2009; Fricano et al., 2012; Sierro et al., 2014). The most widely used intraspecific classification of cultivated tobacco today is based on differences in plant morphology, as well as the quantitative and qualitative composition of their secondary metabolites. These indicators determine the key characteristics of tobacco raw materials, with this classification referred to as “market” (Lewis, Nicholson, 2007). There are eight market classes: Burley, cigar filler, cigar roll tobacco, dark air-cured tobacco, dark steam-cured tobacco, flue-cured tobacco, Maryland, and Oriental tobacco (Moon et al., 2009). Despite the high level of phenotypic variability among cultivated tobacco varieties (Lewis, Nicholson, 2007), the level of nucleotide variability revealed by restriction fragment length polymorphism (RFLP), random amplified polymorphic DNA (RAPD), amplified fragment length polymorphism (AFLP) markers, and genome-wide association studies (GWAS) is relatively low (Brandle, Bai, 1999; Ren, Timko, 2001; Rossi et al., 2001; Tong et al., 2020).

**Fig. 2.**
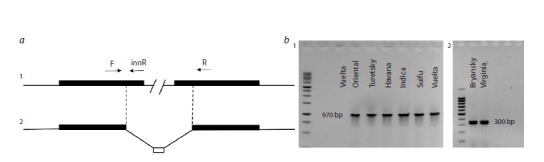
TA cT-DNA in the genomes of N. tabacum а – the schematic representation of TA is shown, with the central part omitted. The orf13 gene is indicated by black rectangles, and the deletion boundaries are marked with dotted lines. The primers selected in the work of G.V. Khafizova and T.V. Matveeva (2020) are also indicated; 1 – TA cT-DNA without deletion; 2 – TA cT-DNA with deletion, where the white rectangle marks a sequence of unknown origin that is 42 bp long; b – fragments obtained using primers F and innR (1), and F and R (2) in PCR analysis of eight N. tabacum varieties (according to (Khafizova, Matveeva, 2020)).

To conduct a phylogenetic analysis and construct a map that accurately reflects the relationships within the species, additional molecular markers are necessary. One such marker is cT-DNA. A study of cT-DNA in the whole-genome data of three N. tabacum varieties revealed an extended deletion in the central part of cT-DNA TA in the Basma/Xanti variety. In contrast, a TA sequence without a deletion was found in the K326 and TN90 varieties (Chen et al., 2014). Differences in the TA structure were previously demonstrated by PCR for the N. tabacum varieties Basma Drama 2, Samsoun, and Xanthi, the cT-DNA of which contains incomplete sequences of the orf13 gene homologue, unlike the cT-DNA in the Wisconsin 38 and Havana 425 varieties, as well as in N. tomentosiformis (Mohajjel-Shoja et al., 2011). Later, eight more varieties of cultivated tobacco were analyzed using the PCR method: Vuelta Abajo, Suifu, Black Indian, Havana 307, Turetsky, Oriental, Bryansky 91, and Virginia × Burley 38 (Fig. 2). The analysis revealed the previously described deletion in the Bryansky 91 and Virginia × Burley 38 varieties, with the deletion localization site coinciding with those in the genomes of the Basma Drama 2, Samsoun, Xanthi, and Basma/Xanti varieties with nucleotide precision (Khafizova, Matveeva, 2020).

The use of the structural variant of cT-DNA as a molecular marker has allowed us to group the varieties belonging to the market class of oriental tobaccos based on the deletion in TA. It is hypothesized that the central part of TA was lost in the ancestral form of N. tabacum, which gave rise to this class. To date, the deletion in TA has been described in only five varieties of cultivated tobacco. Given the limited sample size, it is premature to conclude whether the presence of this deletion serves as a definitive marker for classifying a variety as oriental tobacco. Nevertheless, the results indicate that the structural polymorphism of cT-DNA in N. tabacum varieties can be utilized as one of the molecular markers for studying intraspecific relationships among varieties. As the list of sequenced ctDNA from different cultivated tobacco varieties expands, new structural differences in cT-DNA sequences may be discovered, potentially leading to the development of additional markers for this purpose.

## Fine polymorphism of cT-DNA in phylogenetic studies

Fine polymorphism of cT-DNA with a common origin in the genome (sharing a common localization site) can be utilized to study interspecific variability and reconstruct phylogenetic relationships within a monophyletic group of descendant species from an ancient transformant. For a correct assessment of the completeness of the speciation process in plants, it is essential to evaluate and compare both intra- and interspecific variability for the studied markers. In this context, it has been proposed to reconstruct individual alleles of the studied markers, particularly for cross-pollinated species (Chen et al., 2022). First, let us focus on the methodology of allele separation.

**Research methods**

In scientific literature, allele phasing or haplotype phasing refers to obtaining sequences of DNA fragments located on one chromosome of a pair of homologous chromosomes or, in the case of polyploids, among homeologous chromosomes. Typically, “alleles” refer to shorter fragments, while “haplotypes” pertain to longer sections.

Haplotype phasing provides additional information compared to that derived solely from the consensus genome sequence. Exact haplotype sequences are valuable in a variety of studies, including phylogenetic reconstruction (Tiley et al., 2024) and hybrid studies (Sun et al., 2020).

Various approaches to haplotype phasing have been described (Snyder et al., 2015). For example, one can physically separate fragments of homologous chromosomes using molecular biology methods followed by separate sequencing of the fragments. Bacterial cloning is one such method. Another approach involves separating haplotypes based on genotyping data regarding nucleotide frequencies at polymorphic positions within a population, utilizing various statistical methods (Browning S.R., Browning B.L., 2011). However, the most rapidly advancing approach relies on high-throughput sequencing data. Within this framework, methods can be categorized based on either the assembly of short reads or the mapping of short reads to a reference genome (Zhang et al., 2020). Let us examine the latter case in more detail.

If two polymorphic positions are located within the same sequenced DNA fragment (read from one or both short reads), they belong to the same haplotype. The haplotype sequence can be reconstructed as long as fragments connecting adjacent polymorphic positions are identified. Consequently, the higher the density of polymorphic positions, the greater the overlap between short reads, and the more increased the read depth, the more reliably and extensively haplotype sequences can be reconstructed. These parameters are particularly important when separating polyploid genomes, where more than two haplotypes must be distinguished, some of which may exhibit reduced variance over relatively long stretches (Schrinner et al., 2020).

The indicators mentioned above depend on the sequencing technology used. The best results can be achieved with Hi- Fi technology, which produces long reads of several tens of kilobase pairs (kb) in high quality, with a reading accuracy exceeding 99 % (Wenger et al., 2019). Hi-Fi sequencing enables the assembly of extended haplotype sections, potentially encompassing the entire genome (Tanaka et al., 2023). In contrast, Oxford Nanopore technology can generate very long reads, reaching several million base pairs (bp) in length, but with lower read quality – around 90 % accuracy (Wang Y. et al., 2021). With sufficient sequencing depth, these long reads can also effectively separate extended sections of the genome into haplotypes. Additionally, short reads obtained using Illumina technology can aid in haplotype separation; however, the resulting fragments are limited to conservative regions where read overlaps are insufficient to connect adjacent polymorphic positions

A popular program for haplotype separation is WhatsHap (Martin et al., 2016). It is compatible with reads from all the aforementioned sequencing methods for both diploid and polyploid organisms. The program requires as input a reference genome sequence, a BAM file mapping reads to the reference genome, and a VCF file containing information on polymorphic positions that distinguish the mapped reads from the reference. The separation process yields a modified VCF file that includes information on the assignment of polymorphic positions to haplotypes. From this file, users can obtain data on the lengths and coordinates of the separated fragments (haplotype blocks), retrieve haplotype sequences in FASTA format, incorporate haplotype information into read mapping visualizations, and more.

Alleles can also be separated through the analysis of Sanger sequencing results. When sequencing DNA fragments from heterozygotes, two “peaks” corresponding to specific nucleotides appear on the chromatograms at the same position (Carr et al., 2009; Dehairs et al., 2016; Xie et al., 2019). To deduce the allele sequences of the gene under study, the sequences from each sample can be represented as a vector, where each cell corresponds to a polymorphic position in the gene for the species being examined. Each cell is filled according to the following rules: “11” if the sample contains the most common nucleotide at that position, “00” if it contains the least common nucleotide, and “10” if two different nucleotides occur at that position in the organism’s genome (indicating a putative heterozygote). The resulting vectors can then be assigned to all possible combinations of heterozygous positions “10” while leaving the single-valued positions “11” and “00” untouched. Since there are homozygotes and samples with one allele among the samples, their sequences form a primary pool of alleles that can later be detected in the remaining samples. Each of these alleles in the diploid must correspond to an allele with alternative values in the polymorphic positions, allowing for the identification of a homologous pair for the primary allele (Zhidkin et al., 2023). By sorting through potential combinations of alleles and selecting those with a higher frequency of occurrence, it is possible to determine the genotypes of the samples.

The approaches described above were applied to study the intra- and interspecific variability of plants in the genera Camellia, Vaccinium, and Arachis. Let us explore these examples in more detail.

**cT-DNA polymorphism in Camellia L. species**

The genus Camellia L. belongs to the Theaceae family and includes several economically and culturally significant species. C. sinensis (L.) Kuntze comprises two main varieties: var. sinensis (primarily used for green tea production) and var. assamica (mainly used for black tea production). In the study by K. Chen et al. (2023), 72 species from 12 out of the 14 sections of the genus Camellia were analyzed, revealing at least 12 different cT-DNA insertions. These sequences span a total of 374 kb and contain 47 open reading frames. The identified genes can be categorized into four types: the first includes 19 plast genes, the second contains 6 opine synthesis genes, the third comprises 4 genes encoding tryptophan monooxygenase, and the fourth consists of all other genes with unknown functions. The protein sequences of the cT-DNA genes exhibit varying levels of similarity to known Agrobacterium sequences, with an average similarity of 73.8 % (standard deviation is 12.8 %). The minimum and maximum similarity values are 46 and 92 %, respectively

Notably, some genes homologous to those found in camellias were also identified in various fungal species, including both ascomycetes and basidiomycetes. Internal inverted repeats are present in 7 of the 12 insertions, likely arising from the simultaneous insertion of multiple copies of T-DNA. The differences between these repeats range from 0.05 to 10 % across different fragments. Considering that the repeats were identical at the time of insertion and using the universal substitution rate of 6.5 × 10–9 per position per year (Gaut et al., 1996), along with the number of substitutions between repeats, it is possible to estimate the approximate time of fragment insertion (Haubold, Wiehe, 2001). This time ranges from 0.04 to 7.5 million years ago (Mya) (Chen et al., 2023).

As different phylogenetic lines of the genus evolved, the fragments were inserted at various stages, making them specific to modern taxa at different levels. It was demonstrated that some fragments were lost in certain lines, and the youngest fragments had not yet fully fixed in the populations of their respective species, being present only in some individuals

With known cT-DNA sequences, their presence or absence across different phylogenetic lines of the studied taxon is a convenient feature that can be easily assessed. Thus, the sequence of divergence among sections within a genus, based on the presence of different cT-DNAs, aligns with the phylogenetic tree derived from whole-exome sequencing (Wu et al., 2022). Variations among repeats can also help clarify both the relative and absolute timing of divergence

In the Thea section, only one cT-DNA variety has been described, which was used to elucidate the phylogenetic relationships among the species in this section. This section comprises eleven species (Min, Bartholomew, 2007), including the tea bush C. sinensis (L.) O. Kuntze and various wild tea species. C. sinensis is further divided into the varieties sinensis, assamica, pubilimba, and dehungensis. A phylogeny was constructed for the nine species of the Thea section of the genus Camellia based on one of the aforementioned insertions (Chen et al., 2022). This insertion is 5.5 kb long, includes three genes: acs-like, sus-like, and a rolB-like fragment, and features inverted repeats of 1 kb each at the ends. Sequence analysis revealed that the acs-like and sus-like genes contain stop codons; however, it was noted that in some species, these stop codons arose independently at different positions. The estimated time of the fragment insertion is 7.5 Mya, which roughly coincides with the origin of section Thea at 6.7 Mya (Wu et al., 2022). Therefore, this insertion serves as a suitable marker for this group, which we anticipate to be free of potential biases associated with incomplete lineage sorting.

**Fig. 3.**
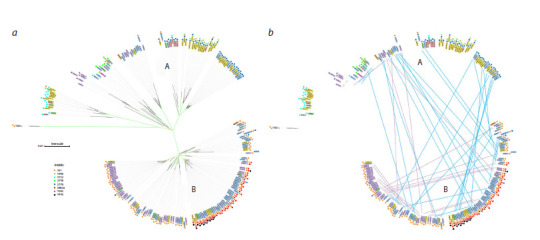
Phylogenetic analysis of the section Thea using cT-DNA. a – phylogenetic tree of 225 clades representing nine species from the genus Camellia, section Thea; b – alleles identified in a single C. sinensis sample are connected by lines. Two main clades are labeled with the Latin letters A and B in both figures. The color designations for the species are as follows: C. sinensis (including C. sinensis var. sinensis, C. pubilimba, C. angustifolia) – blue; C. sinensis var. assamica – purple; C. tachangensis – dark yellow; C. crassicolumna – green; C. gymnogyna – yellow; C. taliensis – pink; C. leptophylla – dark green; C. kwangsiensis – orange; C. ptilophylla – red; C. fangchengensis – no color. Indels are indicated by colored dots. Bootstrap values are color-coded: green – 85–100 %; yellow – 50–85 %; red – less than 20 %. The figure is based on illustrations from (Chen et al., 2022), published under a CC BY 4.0 license.

During the assembly of fragments from short reads, it was observed that many samples contained two alleles, with 2 to 7 % of positions exhibiting polymorphism. Following the analysis of 142 samples, 225 alleles were obtained, forming the basis for constructing the phylogenetic tree (Fig. 3). It was noted that the clades obtained often did not align with species boundaries, and many heterozygous samples contained alleles from different, distantly related clades. Particularly notable in some samples was the presence of alleles from clades separated by a significant evolutionary distance. The maximum divergence observed between alleles from different clades within a single sample reached about 4 %. Based on the premise that such allelic divergence would be unlikely within a single species population, it was suggested that alleles from the major clades evolved within individual ancestral species of the Thea section. This process likely led to the formation of modern species characterized by high allelic diversity due to introgressive crosses. Furthermore, if we assume that the number of major clades corresponds to the number of ancestral species, we can estimate the relationships among these species based on tree topology, divergence time derived from the number of substitutions between alleles, and the contributions of allele frequency to modern populations. For instance, two major pairs of phylogenetically distant clades can be identified, between which hybridization occurred. So, as a result of certain crosses, the species C. sinensis emerged, with one of the presumed ancestral species exhibiting twice as many alleles as the other. The 2.5 % divergence between alleles suggests that these species diverged approximately 1.9 Mya. In contrast, within the second pair, which diverged 2.9 Mya, the species C. tachangensis arose as a result of equally participatory crosses

This work raises important questions regarding the revision of species boundaries, the intensity of interspecific crosses, and other aspects of the evolutionary dynamics within this section.

**Polymorphism of cT-DNA in species of the genus Vaccinium**

The first naturally transgenic species from the genus Vaccinium L. described in the literature was the large-fruited cranberry, V. macrocarpon Aiton (Matveeva, Otten, 2019). Using the BLAST algorithm, a cT-DNA sequence was identified in the complete genome of the cranberry, represented by a single, intact copy of the rolB/C-like gene. This gene belongs to the plast genes, and the amino acid sequence corresponding to it exhibits greater homology with the Plast-proteins of organisms such as Laccaria bicolor (Matre) Orton, Nyssa sinensis Oliver, and Ensifer sp., than with similar sequences from the genomes of known species of agrobacteria. This may suggest that these sequences originated from different transformation events involving the same unknown species of Agrobacterium (Matveeva, Otten, 2019). The second Vaccinium species found to contain a homologue of the studied gene was the highbush blueberry, V. corymbosum (Matveeva, 2021). Subsequently, we demonstrated the presence of a rolB/C-like gene in 26 additional Vaccinium species, as well as in Agapetes serpens (Wight) Sleumer, which belongs to the same family, Ericaceae (Zhidkin et al., 2023). The common localization site of the detected sequences in both Vaccinium and A. serpens is also noteworthy. This wide distribution of the transgene throughout the genus and the shared localization site suggest that the transformation occurred in a common ancestor of the studied species. Consequently, the rolB/C-like gene sequence can be utilized as a marker for reconstructing phylogenetic relationships among these species. This task is particularly relevant, as the phylogeny of the genus Vaccinium remains contentious (Becker et al., 2023)

In all studied species, the rolB/C-like gene is represented by a full-length sequence. The exception is the common cranberry, V. oxycoccos L., where most samples exhibited large deletions of varying lengths in the central part of the gene; however, some samples contained a full-length sequence of the rolB/C-like gene. In the remaining species, polymorphism of the rolB/C-like gene was characterized by single-nucleotide substitutions and indels that are multiples of three, preserving the open reading frame. Furthermore, the pattern of these nucleotide differences was species-specific

Despite the long-standing use of cranberries, blueberries, bilberries, and lingonberries by humans for food and medicinal purposes, the selection of these crops began in the early 20th century (Wang H. et al., 2017; Vorsa, Zalapa, 2019; Sultana et al., 2020). This selection work led to the development of the genus system, the first version of which was established in 1945 (Camp, 1945). The classical system of the genus was based on various morphological features and divided it into sections. Over time, this system was regularly updated and modified, as interspecific hybridization and polyploidization are common in the genus (Camp, Gilly, 1943; Hancock, 2008). These characteristics made it difficult to clearly determine the phylogenetic relationships between species, prompting the development of molecular phylogenetics and the application of DNA barcoding methods to address these issues (Kron, 2002; Powell, Kron, 2003). The dendrogram obtained from the ITS (internal transcribed spacer) and matK (plastid gene of maturase K) sequences contradicted classical ideas about the division of the genus into sections and indicated the polyphyly of the genus Vaccinium. However, using classical phylogenetic markers in cladistic analysis for species where hybridization and polyploidization play significant roles can lead to errors (Soltis, 2002). In contrast, data obtained using SSR markers (simple sequence repeats — microsatellite DNA) (Zalapa et al., 2015; Schlautman et al., 2017), phylogenomics (Diaz-Garcia et al., 2019; Kawash et al., 2022), and chemotaxonomy (Leisner et al., 2017) showed fewer contradictions with classical concepts. Genome sequencing is labor-intensive and expensive, so it has been conducted only on economically significant species. The rolB/C-like gene, as a phylogenetic marker, allowed for the inclusion of more species in the analysis.

In the studied species, the intraspecific variability of the transgene (unlike in species from the Сamellia section Thea) was lower than the interspecific variability, and the mosaicism of some clades could be attributed to hybridization events among the species within those clades. Phylogenetic analysis revealed the unification of representatives from the sections Oxycoccus, Vaccinium, Myrtillus, and Conchophyllum into distinct clades. In contrast, species from the section Cyanococcus did not form a monophyletic group, possibly due to its polyphyletic nature or hybridization events during the development of North American blueberry varieties. The remaining species studied are single representatives of the sections Bracteata, Hemimyrtillus, Vitis-idaea, Oxycoccoides, and Praestantia; therefore, further research is needed. Given the simplicity and low cost of the developed molecular marker, new species can easily be included in subsequent analyses. In other words, the phylogeny of the genus Vaccinium, determined from the sequences of the rolB/C-like gene, shows greater similarity to the traditional classification of the genus than to the phylogeny constructed based on the ITS and matK markers. Results similar to those obtained using the rolB/Clike gene marker have also been reported by other authors employing NGS (next generation sequencing) approaches (Diaz-Garcia et al., 2019; Kawash et al., 2022).

**Polymorphism of cT-DNA in species of the genus Arachis**

The genus Arachis L. comprises 80 species and is divided into nine taxonomic sections: Arachis (with genomes A, B, K), Erectoides (genome E), Extranervosae (genome EX), Procumbentes (genome PR), Caulorrhizae (genome C), Heteranthae (genome H), Rhizomatosae (genome R), Trierectoides (genome TE), and Triseminatae (genome T) (Stalker et al., 2017). Current understanding of the evolutionary relationships among representatives of the genus Arachis relies on morphological, geographical, molecular genetic, and cytogenetic data; however, many controversial issues regarding the structure of the genus remain (Krapovickas, Gregory, 2007; Koppolu et al., 2010; Stalker, 2017; Tian et al., 2021). A. duranensis and A. stenosperma, while A. ipaensis contains the B genome (Matveeva, Otten, 2019, 2021). The list of nGMOs was later expanded to include representatives of the Erectoides, Extranervosae, Procumbentes, Caulorrhizae, and Heteranthae sections (Bogomaz et al., 2024).

In total, 23 naturally transgenic species from this genus are currently known, and there is a high probability that this list will expand in the near future, as the studied nGMOs form a monophyletic group with a common ancestor that was transformed before the studied sections diverged. A homolog of the cucumopine synthase gene (cus) was found in all studied species. In addition to the cus-like gene, B-genome species contained remnants of the mas2′ gene, PR-genome species contained remnants of the mas1′ gene, A. macedoi contained remnants of the ags gene, and A. pusilla contained remnants of both the mas2′ and ags genes (Bogomaz et al., 2024). All of these genes encode enzymes belonging to the same biosynthetic pathway, catalyzing the reactions that lead to the synthesis of agropin, and are found clustered together in the same Agrobacterium T-DNA (Ellis et al., 1984). The common ancestor of Arachis species was likely transformed by a strain containing all three genes; however, at some point, these genes ceased to provide selective advantages to their hosts, accumulated mutations, and were lost, remaining as separate fragments in representatives of different clades

In contrast, the homologue of the cucumopine synthase gene has remained intact in most of the studied species. In cultivated peanut, which is a tetraploid with genomes A and B, the cus-like gene is present in both genomes; in genome A, it is intact, while in genome B, it is mutant (Matveeva, Otten, 2019). More detailed studies showed that among the 29 described alleles of the gene from genome A, only three contained mutations incompatible with its function (Bogomaz et al., 2024). Cultivated peanut is divided into two subspecies: hypogaea and fastigiata (Krapovickas, Gregory, 2007; Bertioli et al., 2011). The most common allele A of the cus-like gene has been identified in representatives of both subspecies, as well as in A. duranensis, confirming their close relationship. However, this allele has not been found in A. monticola, where its other alleles are evenly distributed among separate subclades within the clade containing the A alleles of the cultivated peanut genome and its relatives (Bogomaz et al., 2024). This finding supports previous descriptions of the close relationship between A. monticola and A. hypogaea (Tian et al., 2021). Based on the data obtained using the cus-like gene as a phylogenetic marker, it is possible that the genetic material of A. paraguariensis contributed to the formation of some varieties of A. hypogaea. Additional research confirms a close relationship between A. paraguariensis and A. duranensis, the ancestral species of cultivated peanut (Moretzsohn et al., 2013).

Meanwhile, phylogenetic studies of peanuts based on the cus-like gene indicate that the most distinct clade on the phylogenetic tree is represented by mutant alleles from the B genome. These sequences exhibit faster divergence and are more suitable for research than those subjected to stabilizing selection.

Consequently, studies of the genus Arachis illustrate some limitations in the use of cT-DNA for phylogenetic analysis.

## Phylogenetic relationships of T-DNA genes in Agrobacterium s. lat and nGMOs

In the previous sections, we explored the use of T-DNA as a molecular marker for studying plant phylogeny. This marker can also be utilized to trace the relationships between nGMO cT-DNA and the T-DNA of currently known strains of Agrobacterium s. lat (Suzuki et al., 2002; Matveeva, Otten, 2021). In one of our group’s studies (Matveeva, Otten, 2021), phylogenetic trees were constructed based on individual opine synthesis genes from all known nGMOs as of 2021, as well as from rhizobia strains characterized at that time. The results indicated that in Parasponia andersonii Planch., cT-DNAs containing homologues of the susL gene were obtained from various strains of Agrobacterium s. lat across different evolutionary stages. A similar pattern was observed in Diospyros lotus L. Conversely, cT-DNAs containing homologues of mikimopine synthase were most likely acquired by different species of tobacco (Nicotiana L.) and toadflax (Linaria Mill.) from a single or closely related strain (Matveeva, Otten, 2021). These findings enhance our understanding of the biodiversity of Agrobacterium s. lat and bring us closer to elucidating the mechanisms of host specificity, which is often linked to the structure and functioning of vir genes inherited with T-DNA as part of Ti(Ri) plasmids (Anderson, Moore, 1979). Addressing host specificity is crucial for optimizing plant genetic transformation protocols

## Conclusion

cT-DNA plays a crucial role in elucidating many controversial aspects of phylogenetic studies. The insertions of various T-DNAs mark significant evolutionary events, indicating groups of species that share a common ancestor. Inverted repeats provide insights into the age of this ancestor and help establish the sequence in which independent T-DNAs entered plant genomes. Analyzing the fine polymorphism of cT-DNA, while considering the allelic states of the markers, allows for tracking microevolutionary events and the consequences of hybridization during incomplete speciation. To date, molecular markers based on cT-DNA have been successfully employed in studying the genera Nicotiana, Camellia, Vaccinium, and Arachis. In the genus Nicotiana, the cT-DNA marker facilitated the identification and dating of major evolutionary stages within the section Tomentisae. In Camellia, the primary outcome was a clear demonstration of incomplete speciation within the section Thea. For Vaccinium, the marker helped confirm some classical ideas about the genus’s system that conflicted with ITS-based data but aligned with NGS data from a small sample of species. In Arachis, the study of cT-DNA clearly illustrated the differing evolutionary fates of transgenes with and without stabilizing selection, highlighting some limitations in the marker’s resolving power under strong stabilizing selection pressure.

## Conflict of interest

The authors declare no conflict of interest.
